# A microbial evolutionary approach for a sustainable future

**DOI:** 10.1111/1751-7915.14331

**Published:** 2023-08-21

**Authors:** Lawrence P. Wackett

**Affiliations:** ^1^ Department of Biochemistry, Molecular Biology, and Biophysics University of Minnesota Twin Cities Minnesota USA

## Abstract

With the continued population increase, more sustainable use of water, land, air and chemicals is imperative. Microorganisms will need to be called upon to aid in many sustainability efforts. Prokaryotes are the fastest‐evolving cellular life, and most manipulatable via synthetic biology. Moreover, their natural diversity in processing organic and inorganic chemicals, and their survivability in extreme niches, make them prime agents to enlist for solving many of society's pressing problems.

## INTRODUCTION

Life, from prokaryotes to animals, has largely evolved under environmental conditions imposed by the Earth's natural systems. For >99.9999999% of life's time arrow, organisms survived by responding successfully to their environment. Very recently, some mark the date as 1950, humans have flipped the script and are now controlling the evolutionary environment, both theirs and that of many other creatures. This has led to the widespread idea that the Earth has crossed from the Halocene Epoch, that started ~11,700 years ago, into the Anthropocene Epoch (McCarthy et al., 2023; Witze, 2023). The term “anthropo” stands for human and highlights that this epoch is now human‐activity dominated.

Human impact on the planet is making new evolutionary niches that did not exist for the first 3.7 billion years of evolution. Examples include humans flooding the earth with >100 million new chemicals, and constructing very unnatural environments, like modern buildings. The World Bank estimates that ~56% of the world's human population, 4.4 billion people, live in urban areas (World Bank, 2023). Much of the lives of those 4.4 billion inhabitants are spent immersed in non‐natural environments: sitting in artificial chairs, standing on synthetic‐polymer rugs, breathing air streaming from ventilation ducts, sleeping on artificial foam materials, swimming in pool water solutions of cyanuric acid and hypochlorous acid.

Plants and animals, that people have interacted with since the dawn of humanity, have been significantly impacted by human activities over the last couple centuries, whereas the influence on global microbial populations is less measurable. It is relatively easy to document deforestation, animal extinction and changes in weather patterns that affect all life. Perhaps, the most observable changes for prokaryotes are enhanced cyanobacterial ocean blooms, readily observable via satellite imagery (Dai et al., 2023). Viruses are even less observable but are clearly felt by humans with the spread of pathogens; for example, bird flu epidemics or the recent SARS‐CoV2 human pandemic.

The present review discusses the need for a better understanding of the utility for microbes to mitigate against damaging changes to planet Earth. While plants and animals are highly susceptible to the changes we have wrought in ocean pH, land desiccation and atmospheric warming, many microbes easily handle extremes of pH, desiccation and temperature. Animals are highly impacted by low molecular weight chemical pollutants and polymers. For these same chemicals, select microbes flourish by being adept in biodegrading and recycling them. With the exception of a limited type of microorganisms harnessed for foods, industrial processes and chemicals, most microbes do their work largely unseen. Nonetheless, they are surely felt by the planet. For example, the global nitrogen cycle is dependent on prokaryotes, as only prokaryotes are known to fix atmospheric nitrogen and return dinitrogen gas from the atmosphere back into the biosphere (Rees & Howard, 2000).

The following sections deal with an inventory of microbial tasks that must be better harnessed to rescue society, and the planet. If carried out, perhaps microbes will need to share the spotlight in Anthropocene 2.0.

## WATER SANITATION

Municipal water treatment was essential for urbanization and, since the late nineteenth century, focused on removing pathogens that spread through water (Sedlak, 2014). The introduction of water chlorination was a major innovation for killing bacteria and viruses in municipal water distribution. But many municipal water treatment systems have not incurred major changes during the past century. Needs have changed. Today, concerns have been raised about the influx of pharmaceuticals and persistent chemicals like poly‐ and perfluoroalkyl substances (PFAS) into wastewater (Sedlak, 2014). Antibiotics entering wastewater may select for tolerant bacteria and contribute to the spread of antibiotic resistance traits (Kalli et al., 2023).

The microbiomes of wastewater treatment plants (WWTPs) have come under increasing study via metagenomic analyses coupled to advances in analytical chemistry (Fenner et al., 2021; Loos et al., 2013). However, WWTPs harbour more complex microbial communities than a typical human gut microbiome, rendering the metabolic networks for entering chemicals to be similarly complex. Creative methods are needed. Recently, a proposal was made to view open water treatment systems via overhead imaging (Moran & Tikhonov, 2022). The idea is based on the proposition that the high complexity of the WWTP microbiome makes for rapid wholesale changes that show up as observable colour, turbidity, foaming and other changes temporally, allowing a coarse‐grained tracking of the system. In another approach, machine learning methods are being applied to microbiome management for WWTPs (Cai et al., 2021; Yu et al., 2023).

### Better land utilization

Better land utilization is a multi‐faceted issue and innovations in microbiology can help in numerous ways (Banerjee & Van der Heijden, 2023; Tripathy et al., 2023). Climate change is already redistributing rainfall patterns and some agricultural areas are expected to experience significant drought conditions in the coming decades. There is already focus on breeding more drought‐tolerant plants. Microbial studies on desiccation stress contribute to our knowledge of how living things deal with a lack of sufficient water. Additionally, plant microbiome composition will play a role in plant water and nutrient acquisitions.

There is a clear understanding that a shift in global diet away from animal protein and more towards plant and microbial substitutes will have many positive impacts on land use and greenhouse gas emissions. Cell culturing methods will need to evolve beyond our classic stirred batch fermentations to help meet the needs for increased sources of plant and microbial nutrition. One largely neglected field is solid‐state fermentation, an art practised for centuries, but not much expanded in scope. Traditional large‐scale fermentations will have significant needs for water and solid‐state fermentation technology will require less water, space and overall capital investment. Solid‐state fermentations have traditionally been most significant for food production and enhancement (Boukid et al., 2023). There are also important applications in converting wastes to products (Zhou et al., 2022). However, full life cycle analyses are required to determine if true improvements can be realized with diminished use of resources (Javourez et al., 2022).

While microbes fix large quantities of atmospheric dinitrogen to make biologically usable ammonia, it is only directly coupled to plant growth in a limited number of crops used for human consumption, such as soybean (Xu & Wang, 2023). The major crop staples of corn, wheat and potatoes require vast inputs of nitrogen derived from the industrial Haber‐Bosch process. The Haber‐Bosch process is essential to support modern agriculture but is energetically inefficient (Cherkasov et al., 2015). The process consumes several percent of the world's natural gas output and contributes similarly to carbon dioxide in the atmosphere. Microbial dinitrogen fixation has long been viewed as having the potential for supplying nitrogen for agriculture more broadly. However, the nitrogen fixation (*nif*) operon is complex, and the large ATP requirement to drive dinitrogen reduction selects against nitrogen fixation when ammonium ion is already present (Dixon & Kahn, 2004; Hardy & Burns, 1968). The general approaches have been to express *nif* genes directly in plant plastids or mitochondria (Bennettt et al., 2023). Other efforts have used plant root associated bacteria and engineered symbiosis to obtain some degree of nitrogen fixation in the soils of cereal crops (Haskett et al., 2022).

## CHEMICAL RECYCLING

Carbon from petroleum is largely a one‐way street moving from extraction in the ground to processing to utility to disposal and, in some cases, biodegradative transformation to carbon dioxide. Many polymers are merely landfilled, not recycled and not biodegraded. This will need to change. Plastic recycling is imperative for preserving environments, minimizing human exposure to microplastics and lowering petroleum consumption by recycling plastic components (Schneiderman & Hillmyer, 2017).

A prime example of plastic recycling is with polyethylene terephthalate (PET), a major polymer used for beverage bottles. A commercial process for PET recycling is based on enzymatic degradation by PETase (Tournier et al., 2020). Commercial PETase enzymes are derived from nature's cutinase enzyme that evolved to hydrolyse a waxy polymer, cutin, on plant leaves (Austin et al., 2018). The enzymatic process is used to recover the terephthalate for recycling into new polymeric material (DeFrancesco, 2020). PET is an ester and is amenable to enzymatic hydrolysis. Other polymers such as polyethylene are less amenable to biodegradation.

Chemical recycling during manufacture is also a target for saving carbon, pollution and money for the manufacturer. An excellent example is the enzymatic processing of 1,2,3‐trichloropropane, a waste product during the large‐scale industrial synthesis of epichlorohydrin (Bosma et al., 2002; Gray et al., 2003). Given that 1 billion pounds of epichlorohydrin was produced, the waste amounted to 30 million pounds and had to be landfilled. A *Rhodococcu*s dehalogenase was identified to transform 1,2,3‐trichloropropane to hydroxydichloropropane that could be treated with NaOH to recycle the chemical to the target chemical, epichlorohydrin. Waste to product is an ideal and shows the promise of meshing microbial enzymes with large‐scale chemical processes for atom efficiency and recycling.

Other examples exist by which inefficient industrial processes can be upgraded through the use of microorganisms and/or their enzymes. For example, phosphogypsum may be upgraded to obtain elemental sulfur and recover more phosphate necessary for agriculture using sulfur‐transforming bacteria (Bounaga et al., 2022). In another example, large resources are invested in making chemical catalysts; for example, those using palladium. A recent report indicates promising results in using bacteria to make upgraded metallic nanoparticles that can significantly enhance the properties of palladium catalysts (Egan‐Morriss et al., 2021). In another example, the mining of lanthanide elements required for electronic devices requires industrial processes that degrade the environment (Chakhmouradian & Wall, 2012). Recent findings that microbes specifically acquire and utilize various lanthanide elements is leading to investigations into more environmentally friendly bio‐recovery processes (Daumann et al., 2022).

## EVOLUTION

Evolution happens. And with a global population of ~10^31^ prokaryotes, it is happening every second of every day, naturally (Whitman et al., 1998). Humans use microbial evolution, but crudely. For example, over the last century, the petroleum and chemical industries have widely used biotreatment ponds in which the chemicals biodegrade via some unknown consortium of microbes (Atlas & Raymond, 1977). The sludge from one pond may be used to inoculate another. This often works well enough. However, as population and chemical needs grow, and as we seek to biodegrade highly persistent molecules such as PFAS, innovation is needed.

Laboratory adaptive evolution has become more standardized recently with laboratory automation, computerization and genome sequencing of improved variants (Sandberg et al., 2019). A Nobel Prize in chemistry was awarded several years ago for developments in a method commonly known as directed evolution (Arnold, 2018). These newer developments are an extension of earlier improvements via chemical mutagenesis and selection or screening. This type of protocol was very useful for obtaining fungal strains that produced higher levels of penicillin in the 1940s, and was also used for bacterial antibiotics (Rowlands, 1984). More recently, antibiotic discovery and improvement has taken advantage of the revolution in microbial genome sequencing and the tools for identifying natural product biosynthetic clusters (Medema & Fischbach, 2015).

Conde‐Pueyo and colleagues have proposed using our increasing knowledge of microbial evolution for combating large‐scale system changes, such as climate change (Conde‐Pueyo et al., 2020). The idea springs from the concept of terraforming Mars and other planets, but may have more immediate application in modifying microbial communities upon our own planet. Currently, human‐evolved microbes are largely used in manufacturing facilities to make bioproducts. Biomanufacturing is clearly increasing and can help make some products with less energy, materials and waste products (Fashkhami & Jarboe, 2023). However, there is a scale between the laboratory and the planet for using accrued knowledge of microbial evolution to help solve societal problems. We should increasingly look to use human‐evolved microbes to handle local environments such as those impacted by hazardous wastes. It is intriguing to consider that microbes can be engineered to explore innovation in such an environment under a desired selective pressure regime that is beneficial to society and the planet (de Lorenzo, 2022). Can we dare to do that? Can we dare not to?

## AUTHOR CONTRIBUTIONS

The conceptualization and writing is the responsibility of Lawrence P. Wackett.

## CONFLICT OF INTEREST STATEMENT

The author declares no conflict of interest regarding the content of this material.
